# Editorial for the Special Issue on 3D Printed Microfluidic Devices

**DOI:** 10.3390/mi9110609

**Published:** 2018-11-21

**Authors:** Savas Tasoglu, Albert Folch

**Affiliations:** 1 Department of Mechanical Engineering, University of Connecticut, Storrs, CT 06269, USA; 2 The Connecticut Institute for the Brain and Cognitive Sciences, University of Connecticut, Storrs, CT 06269, USA; 3 Department of Biomedical Engineering, University of Connecticut, Storrs, CT 06269, USA; 4 Institute of Materials Science (IMS), University of Connecticut, Storrs, CT 06269, USA; 5 Institute for Collaboration on Health, Intervention, and Policy (InCHIP), University of Connecticut, Storrs, CT 06269, USA; 6 Department of Bioengineering, University of Washington, Seattle, WA 98195, USA

Three-dimensional (3D) printing has revolutionized the microfabrication prototyping workflow over the past few years. With recent advances in 3D printing and 3D computer-aided design (CAD) technologies, highly complex microfluidic devices can be fabricated via single-step, rapid, and cost-effective protocols as a promising alternative to the time-consuming, costly, and sophisticated poly(dimethylsiloxane) (PDMS) molding and traditional cleanroom-based fabrication. Microfluidic devices have enabled a wide range of biochemical and clinical applications, such as cancer screening, micro-physiological system engineering, high-throughput drug testing, and point-of-care diagnostics. Using 3D printing fabrication techniques, the alteration of design features is significantly faster and easier than with traditional fabrication, enabling agile iterative and modular design, 3D printing services and marketplaces, and rapid prototyping. Biocompatible resins for 3D printing are now available that, contrary to PDMS, feature very low drug absorption and are thus very good material candidates for building complex 3D organ-on-a-chip systems in the future. These advances will make microfluidic technology more accessible to researchers in various fields and will accelerate innovation in the field of microfluidics. Accordingly, this Special Issue showcases 14 research papers, communications, and review articles that focus on novel methodological developments in 3D printing and its use for various biochemical and biomedical applications. The papers of this special issue explore the following aspects of 3D printing and how it pertains to microfluidic devices: (1) fabrication methods and materials, (2) applications for equipment and tools, (3) biological applications, and (4) biological compatibility.

1. Fabrication methods and materials: Fuad et al. [1] characterized additively manufactured molds fabricated via stereolithography and material jetting, as well as the positive replicas produced by soft lithography and PDMS molding. They showed that stereolithography provides finer part resolution with no toxicity observed in the corresponding positive replicas. Beauchamp et al. [2] demonstrated a custom 3D printer which, via optical dosage control, provides very high-resolution printing capabilities down to ~30 µm and ~20 µm scales for positive and negative surface features, respectively. The custom printer was used to fabricate and optimize various microfluidic particle traps. Kim et al. [3] also developed a novel printing scheme: specifically, a sequential stereolithographic co-printing process which utilizes two different molecular weight poly(ethylene glycol) diacrylate (PEG-DA) resins to produce microchannels with embedded porous barriers. The semi-autonomous fabrication process reduced the processing time, manufacturing costs, and eliminated complications with assembly. In addition to fabrication methods, Kotz et al. [4] presented work on materials formulated for 3D printing, namely highly fluorinated perfluoropolyether (PFPE) methacrylates, for which 3D printing has seldom been demonstrated. The developed formulations, printed and cured using stereolithography, exhibited high light transmittance and high chemical resistance to organic solvents.

2. Applications for equipment and tools: Brennan et al. [5] utilized the capabilities of 3D printing technology to create an open source design for micropipettes which can be assembled from 3D-printed parts and a disposable syringe. The open source design exemplifies scientific tools that can be produced via 3D printing as inexpensive alternatives to commercial products. Oh et al. [6] also developed 3D-printed laboratory equipment to measure the viscosity of fluids, which is applicable, for example, to the quality assurance of liquid products and for monitoring the viscosity of clinical fluids. They designed 3D-printed capillary circuits, with graduations to serve as a flow meter for easy readability and a syringe modified with an air chamber to generate pressure-driven flow, to provide equipment and calibration-free viscosity measurement of Newtonian and non-Newtonian fluids. Van den Driesche et al. [7] presented methods for a wide variety of applications to design and fabricate microfluidic chip holders with integrated fluidic and electric connections, such as fluidic sealing by O-rings and electric connections by spring-probes without glue or wire bonding. Microfluidics can also be applied to 3D printing technologies, as demonstrated by Serex et al. [8]. For application to bioprinting, Serex et al. demonstrated the integration of micromixers, micro-concentrators, and microfluidic switches into the tip of the print heads for extrusion-based 3D printing, thereby enabling new prospects in 3D bioprinting.

3. Biological applications: There are countless biological applications for 3D-printed microfluidic devices, a few of which are included in this Special Issue. Lim et al. [9] reported an automated platform for a colorimetric malaria-Ab assay, assembled from stereolithographic-printed elastomeric reservoirs, fused deposition modeling-printed framework, plastic tubing, servomotors, and an Arduino microcontroller chip. Kim et al. [10] demonstrated a 3D-printed millifluidic platform for bacterial preconcentration and genomic DNA (gDNA) purification, by immunomagnetic separation and magnetic silica-bead-based DNA extraction, to improve the molecular detection of pathogens in blood samples. The platform was verified for preconcentrating E. coli in blood, suggesting that the platform is a useful tool for lowering limitations on molecular detection. In addition to these research articles, Sharafeldin et al. [11] wrote a review on the applications of 3D-printed microfluidic devices in biomedical diagnostics and on how 3D printing enables low-cost, sensitive, and geometrically complex devices. Three-dimensional printing can be used for the fabrication of microfluidics, supporting equipment, optical and electrical components, in addition to 3D bioprinting which can incorporate living cells or biomaterials into diagnostic systems.

4. Biological compatibility: In their work on connections and holders for microfluidic devices, for bioanalysis applications, van den Driesche et al. [7] addressed the possible cytotoxicity of cured 3D-printed resin by introducing a surface coating of parylene-C. Carve et al. [12] reviewed commonly used vat polymerization and material jetting materials with respect to the materials’ biocompatibility, in addition to discussing methods to mitigate material toxicity to promote the application of 3D-printed devices in biomedical and biological research, such as for monolithic lab-on-chip devices. In addition to biocompatibility with cells, interactions with biomolecules such as protein have been studied by Lepowsky et al. [13]. They demonstrated a simple cleaning chip design with an integrated cleaning procedure to study the long-term cyclic biofouling burden on 3D-printed microfluidic devices, and verified the cleaning chip for urine sampling handling for a protein assay. Lepowsky et al. [14] also provided a perspective on traditional and emerging anti-fouling methods as applicable to enabling the greater reusability of 3D-printed microfluidic devices for biomedical applications.

We wish to thank all authors who submitted their papers to this Special Issue. We would also like to acknowledge all the reviewers for dedicating their time to provide careful and timely reviews to ensure the quality of this Special Issue.
